# Neurogenic and Neuroprotective Potential of Stem/Stromal Cells Derived from Adipose Tissue

**DOI:** 10.3390/cells10061475

**Published:** 2021-06-11

**Authors:** Anna Figiel-Dabrowska, Klaudia Radoszkiewicz, Paulina Rybkowska, Natalia Ewa Krzesniak, Dorota Sulejczak, Anna Sarnowska

**Affiliations:** 1 Translational Platform for Regenerative Medicine, Mossakowski Medical Research Institute, Polish Academy of Sciences, 02-106 Warsaw, Poland; adabrowska@imdik.pan.pl (A.F.-D.); kradoszkiewicz@imdik.pan.pl (K.R.); prybkowska@imdik.pan.pl (P.R.); 2 Department of Plastic and Reconstructive Surgery, Centre of Postgraduate Medical Education, Prof. W. Orlowski Memorial Hospital, 00-416 Warsaw, Poland; natalia.krzesniak@wp.pl; 3 Department of Experimental Pharmacology, Mossakowski Medical Research Institute, Polish Academy of Sciences, 02-106 Warsaw, Poland; dsulejczak@imdik.pan.pl

**Keywords:** ASC, DFAT cells, neurotrophic factors, oxygen–glucose deprivation injury, neural differentiation, neuroprotective potential, organotypic hippocampal slices, indirect co-culture, low oxygen concentration

## Abstract

Currently, the number of stem-cell based experimental therapies in neurological injuries and neurodegenerative disorders has been massively increasing. Despite the fact that we still have not obtained strong evidence of mesenchymal stem/stromal cells’ neurogenic effectiveness in vivo, research may need to focus on more appropriate sources that result in more therapeutically promising cell populations. In this study, we used dedifferentiated fat cells (DFAT) that are proven to demonstrate more pluripotent abilities in comparison with standard adipose stromal cells (ASCs). We used the ceiling culture method to establish DFAT cells and to optimize culture conditions with the use of a physioxic environment (5% O_2_). We also performed neural differentiation tests and assessed the neurogenic and neuroprotective capability of both DFAT cells and ASCs. Our results show that DFAT cells may have a better ability to differentiate into oligodendrocytes, astrocytes, and neuron-like cells, both in culture supplemented with N21 and in co-culture with oxygen–glucose-deprived (OGD) hippocampal organotypic slice culture (OHC) in comparison with ASCs. Results also show that DFAT cells have a different secretory profile than ASCs after contact with injured tissue. In conclusion, DFAT cells constitute a distinct subpopulation and may be an alternative source in cell therapy for the treatment of nervous system disorders.

## 1. Introduction

The application of stem cell therapy in regenerative medicine of the nervous system and neurodegenerative diseases gives much promise today, but its application is still insufficiently proven. The ability to assume not only the phenotypic but also the functional character of neural cells, such as the secretion of neurotrophic factors, is a fundamental determinant of the quality and effectiveness of cell therapy. Obtaining such neuro-specialized stem cells is currently a challenge. The ability of stem cells to differentiate into neuronal cells has been demonstrated in many in vivo studies using induced pluripotent stem cells (iPS) [1] and embryonic stem cells (ESC) [2], but in clinical use there are many obstacles to overcome, and the risk may exceed the therapeutic benefit [3].

Due to the current use of liposuction, which is considered as a minimally invasive method of tissue isolation, adipose tissue still remains the most promising source of stem/stromal cells. After tissue isolation and collagenase digestion, the stromal vascular fraction (SVF) can be obtained. After plating the cells and cultivation, adipose-derived stem/stromal cells are obtained. The two described cell populations derived from adipose tissue have different compositions and properties and should not be equated with each other [4,5]. Currently, nearly 500 clinical trials worldwide (including: SVF- stromal-vascular fraction, ASC—adipose-derived stem/stromal cells) are assessing the therapeutic properties of adipose-derived regenerative cells in various diseases. Their main application is in aesthetic medicine in the form of adipose tissue transplantation, in which the regenerative effect could be enhanced by SVF. Additionally, ASCs are widely used in orthopedics as a treatment of bone and musculoskeletal system defects as well as in oral surgery bone reconstruction/regeneration. However, more and more studies are being based on ASC immunomodulatory and/or trophic properties, including Crohn’s disease and autoimmune diseases regarding the nervous system [6]. Nevertheless, clear and credible evidence of ASC efficacy in the treatment of neurological diseases has not been adequately confirmed, especially ASCs’ capacity to differentiate toward neural cells. This ability is disputable and explained by the presence of a sparse pluripotent-like cell subpopulation. ASCs are a highly heterogenic population of cells, and their composition could depend on the individual variability of donors, such as age, sex, or coexisting diseases, as well as on the isolation technique, but the main reason could also be the origin of isolated fractions, which remains unknown [7,8]. Therefore, one of the directions for further investigation is to look at the distinction of the adipose-derived cell population and to select the one that will exhibit the most pluripotent features or potential to differentiate toward neuronal cells.

Considering the basic processes underlying neuronal differentiation, attention should be paid to the physiological epithelial–mesenchymal transition mechanism (EMT) during the embryogenesis. In early vertebrate development, neural crest cells undergo an epithelial–mesenchymal transition, delaminate, and migrate to form a variety of tissues and cells throughout the body. They differentiate into neurons, glial cells, connective tissue, melanocytes, and cartilages [9]. It is possible that about 2% of ASCs’ subpopulations of cells isolated from the heterogeneous fraction of adipose tissue and that the origin is not fully elucidated and may be a remnant of the epithelial–mesenchymal transition of nerve crest cells. Thus, the neuro-ectoderm may be the origin of some subpopulations of cells found in the adipose tissue stromal cell fraction (NCDASCs) [10]. Thus, the separated subpopulation could be a promising candidate for cells with greater neuronal differentiation capacity, but their ability to differentiate into functionally mature neurons was not observed [11].

One of the methods for obtaining an adipose tissue subpopulation with increased pluripotent-like features was proposed by Yagi et al. He for the first time found and characterized a novel pre-adipocyte cell subpopulation established from mature adipocytes of adult mice [12]. These reports were also confirmed by Matsumoto et al., proving that such a subpopulation has multilineage potential and can also be separated from human adipose tissue derived from liposuction [13]. Cells that have a fibroblast-like phenotype and maintain proliferation ability are referred to as dedifferentiated fat cells (DFAT). The origin of DFAT cells is not fully understood; however, it is believed that mature adipocytes are forced to dedifferentiate by a ceiling culture procedure and to revert to a more primitive phenotype [14]. There are numerous promising reports of DFAT cell transplantation in injured tissue areas in animals. Their positive therapeutic effect has been shown in the promotion of recovery from spinal cord injury in rats [15], the regeneration of nerves in an experimental rat facial nerve defect model [16], the alleviation of brain damage in the neonatal hypoxic-ischemic encephalopathy model [17], and the neovascularization in a mice hind-limb model of ischemia [14]. Those findings may suggest that DFAT cells could be a promising substitute in regenerative stem-cell based therapies in neurological injuries and could have a great ability for clinical application. Therefore, we decided to use DFAT cells and to further define their neurogenic and neuroprotective properties in vitro. We implemented several techniques to ultimately support these abilities, optimizing the conditions previously used on ASCs culture.

In this study, we investigated and compared the neuroprotective properties of both populations of the stem/stromal cells derived from adipose tissue, related to their paracrine activity as well as to their neurogenic capacity in response to the presence of intact or injured nerve tissue due to oxygen–glucose deprivation (OGD) using an organotypic rat hippocampal slice culture model (OHC). We believe that understanding the “stem/stromal cell–neural tissue” interactions can provide insight on the restorative abilities of the CNS.

**Figure 1:**
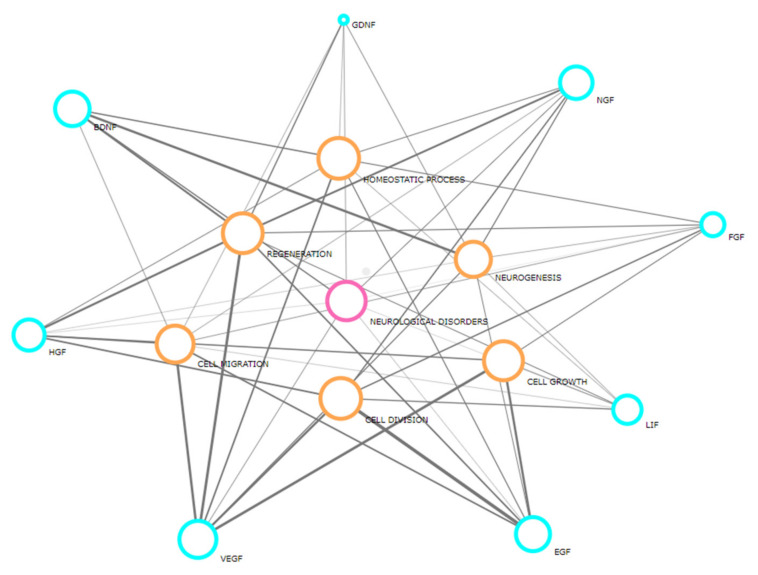
The correlation between processes (orange circles) and factors (blue circles) chosen for the analysis in this study. Created with Biovista Vizit (https://www.biovista.com/vizit/, accessed date: 17 November 2020).

At present, we know that the profile of stem cell secretome, which originates from different sources, varies in response to environmental changes [18,19,20,21,22,23]. In the adult human brain, neurogenesis depends on the presence of endogenous and exogenous substances responsible for proliferation, survival, and the ability to differentiate in stem cell niches [24,25]. The transplantation of adipose-derived stem/stromal cells offers trophic support for neural cells. Potential benefits of ASCs’ therapeutic use were previously shown to be mainly due to their secretive abilities by promoting the survival of the endogenous cells and suppressing the inflammatory response [26]. Moreover, ASCs can activate and support endogenous neurogenesis by secreting numerous growth factors. The results of in vitro and ex vivo experiments showed that ASCs increased the secretion of brain-derived neurotrophic factor (BDNF), glial cell-derived neurotrophic factor (GDNF), vascular endothelial growth factor-A (VEGF-A), basic fibroblast growth factor (bFGF), leukemia inhibitory factor (LIF), and insulin-like growth factor (IGF) [27,28]. Other neurotrophic factors that were reported to be secreted by those cells include epidermal growth factor (EGF), nerve growth factor (NGF), and hepatocyte growth factor (HGF) [24,29,30,31]. The correlation between processes and described factors is presented in the Figure 1. We analyzed the concentration levels of each of those factors in the media from the basic ASCs/DFAT cell culture, the co-culture of ASCs/DFAT cells with OHC, and with OHC after OGD.

As the ability for the neuro-restoration is still the domain of the future, current priorities refer to the selection of the appropriate environmental conditions of the stem/stromal cells culture that is conducive to their neural differentiation [32]. Therefore, our goal was to determine the culture conditions stimulating the neural differentiation that can simultaneously allow the maintenance of the cells’ high proliferation rate.

Despite the initial doubts about the ability of those cells to differentiate into non-mesodermal cells, several research studies indicate that the cells’ neural differentiation properties in vitro is in response to the presence of specific factors [33,34,35]. Such stimulating factors are neurotrophins, hormones, and growth factors. In our experiments, we decided to use basic fibroblast growth factor (bFGF), which during recent years of studies was shown to stimulate neural differentiation. There are single reports demonstrating the beneficial effect of bFGF on ASCs’ differentiation into early neural progenitors [36,37,38,39,40]. Another treatment that we chose for inducing neural differentiation in our experiments was the inhibitor of the Wnt pathway, retinoic acid (RA). Similar to bFGF, RA has been reported to be an effective stimulator of ASCs’ neural differentiation in combination with other factors and as a single component of the differentiating medium [38,41,42,43].

The alternative to the single factors presented above is suggested by several protocols that propose the use of chemical compound “cocktails”. There are attempts to use mixes containing factors stimulating neuronal differentiation, such as forskolin, insulin, hydrocortisone, and valproic acid in the media [25,44], ongoing both with the maintenance of the cells’ high proliferation rate. In this study, we analyzed the impact of one of them that was not previously used in ASCs’ differentiation, commercially available supplement N21. Interestingly, we found the results of this culture variant to be the most satisfying; thus, we applied it in the following studies assessing the potential of neurogenic DFAT cells.

**Figure 2:**
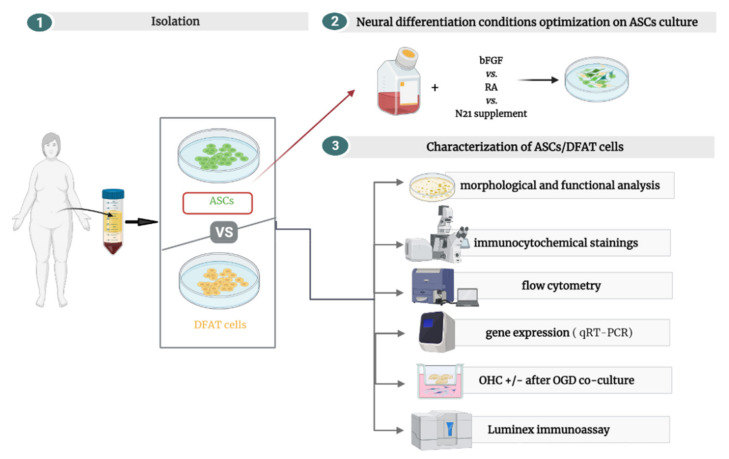
General overview of our study experimental steps. After isolation, ASCs were used to optimize the best conditions for neural differentiation, which were applied in further investigation of ASCs/DFAT cells’ proliferative, neurogenic, and neuroprotective potential. The figure was created in BioRender (https://biorender.com/, accessed date: 10 January 2021).

A general overview of the study steps is presented in the Figure 2.

## 2. Materials and Methods

### 2.1. Cell Isolation

**Figure 3:**
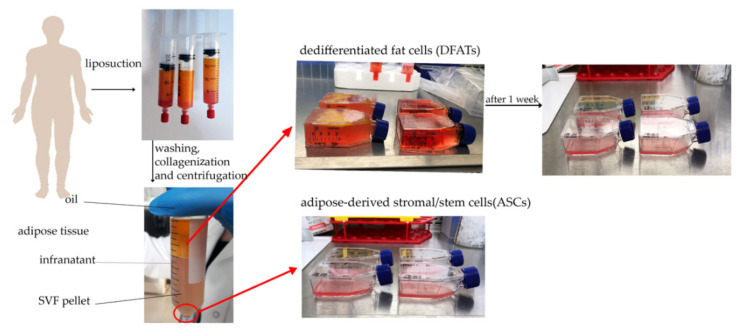
Steps of ASCs and DFATs cell isolation procedure. ASCs cultured as a standard monolayer and DFAT cells cultured using ceiling method. After one week, DFAT cells were reverted and treated with the same culture conditions (5% O_2_, 5% CO_2_, 37 °C) as ASCs.

Adipose tissue was collected during the liposuction procedure from 4 patients (2 males, 2 females, 44−74 years old ± SD) of the Plastic Surgery Department at Orlowski’s Clinical Hospital in Warsaw. The study was conducted according to the guidelines of the Declaration of Helsinki, and approved by the Bioethical Committee at the Centre of Postgraduate Medical Education (No. 62/PB/2016) on 14 September 2016. All patients (participants of the study) signed an informed consent to participate in the study. The subcutaneous abdominal fat tissue was filled in 50 mL Falcon tubes (Beckton Dickinson, Franklin Lakes, NJ, USA) and digested with type VI GMP Grade collagenase (Serva, Heidelberg, Germany). After a series of centrifugations, supernatant collections, and washing with PBS (Macopharma, Tourcoing, France), the SVF and digested fat tissue were used in the next step of the procedure (Figure 3).

Stromal cells fraction was collected and suspended with 5 mL of basic growth medium and next transferred into 25 cm^2^ flasks (Nunc, Thermo Fischer Scientific, Waltham, MA, USA) to perform standard ASCs culture.

In order to obtain DFAT cells, a respective floating layer, which was a mature adipocyte layer, was transferred into 25 cm^2^ flasks (Nunc, Thermo Fischer Scientific) filled completely with basic growth medium. Such prepared cell culture was carried out using the ceiling method for one week, then the obtained DFAT cells were processed with the same methods as ADSC.

### 2.2. Cell Culture

The obtained cells were cultured in basic growth medium: MEM-alpha (Macopharma, Tourcoing, France), human platelet lysate (10%, Macopharma, Tourcoing, France), penicillin/streptomycin (1%, Gibco, Thermo Fisher Scientific, Waltham, MA, USA), and heparin (0,1%, Sigma-Aldrich, Saint Louis, MO, USA) in humidified incubators under 21% O_2_ and 5% CO_2_ at 37 °C. The culture medium was replaced every 2/3 days, when cell culture was subconfluent; cells were detached from the dishes with accutase (Accutase Cell Detachment Solution, Beckton Dickinson, Franklin Lakes, NJ, USA).

### 2.3. Flow Cytometry Analysis

The expression of specific surface markers was analyzed with Human MSC Analysis Kit (Beckton Dickinson). According to manufacturer’s protocol, the cells were detached with accutase (Beckton Dickinson) and suspended in BD Pharmigen Stain Buffer (Beckton Dickinson), then the specific fluorochrome-conjugated antibodies against mesenchymal stem cells positive markers (CD90, CD73, CD105) and negative markers (CD34, CD11b, CD19, CD45, HLA-DR) were added. Samples were incubated in the dark at room temperature for 30 min. After the incubation, cells were centrifuged, washed, and suspended in a buffer according to the protocol. The samples were analyzed immediately using FACSDiva software (Beckton Dickinson). The results are presented as histograms.

### 2.4. Mesodermal Lineage Differentiation

The cells’ osteogenesis, adipogenesis, and chondrogenesis abilities were investigated using the cells (at 3rd passage) that were seeded on 24-well plates (Nunc, Thermo Fischer Scientific) in 10^4^ cells/cm^2^ density and cultured in commercial differentiation medium (Gibco, Thermo Fisher Scientific, Waltham, MA, USA). After 14 days of adipogenic and chondrogenic differentiation and 21 days of osteogenic differentiation, the cells were fixed in 4% PFA, stained as listed in Table 1, and closed with Fluorescent Mounting Medium (Sigma-Aldrich). The results were analyzed with Axio Vert.A1 (Carl Zeiss, Oberkochen, Germany) inverted microscope and ZEN software (Carl Zeiss, Oberkochen, Germany).

### 2.5. Cell Proliferation Analysis

Long-term cell proliferation analysis was performed by indication of the population doubling time (PDT), which is based on the total cell number at each passage. To calculate PDT, the following formula was used: PDT = (t − t_0_) × log _2_/(log N−log N_0_), where: t − t_0_ is the duration of passage (days), N is the number of cells from passage/1 cm^2^, and N_0_ is the number of harvested cells/1 cm^2^.

### 2.6. Senescence Assay

The cells at 3rd and 8th passage were seeded at a density of 3000 cells/cm^2^ on 6-well plates (Nunc, Thermo Fischer Scientific) and cultivated in standard condition due to subconfluency. Then, senescence analysis was performed with a Senescence Cells Histochemical Staining Kit (Sigma-Aldrich) according to the manufacturer’s protocol. Shortly thereafter, the cells were washed with PBS (Macopharma), fixed with fixation buffer, and stained with staining mixture. Then, the cells were incubated in 37 °C and 1% CO_2_ concentration overnight. On the following day, the cells were fixed with 4% PFA (Sigma-Aldrich) and counted. The percentage of positive β-galactosidase cells was calculated based on the number of stained cells and total number of cells.

### 2.7. CFU-F Assay

The cells at 3rd and 8th passage were seeded on 6-well plates (Nunc, Thermo Fischer Scientific) at a density of 10 cells/well and cultured in basic growth medium for 10 days. Then cells were fixed with 4% PFA for 15 min and stained with 0.5% toluidine blue (Sigma-Aldrich) for 20 min, rinsed once with distilled water, and the number of stained colonies (with more than 50 cells) was counted. CFU frequency was calculated as the number of colonies per number of seeded cells.

### 2.8. Neural Lineage Differentiation

Cells’ capability of neural lineage differentiation was evaluated in two ways. Firstly, neurogenesis was induced with modification of medium composition. The cells at the 3rd passage were seeded at a density of 3000 cells/well on 24-well dishes (Nunc, Thermo Fischer Scientific) and cultured in humidified conditions under 21% O_2_, 5% CO_2_ at 37 °C in medium that consisted of:

- cell growth medium with Human Platelet Lysate concentration decreased to 5% and with the addition of bFGF (0.1%; Gibco) for 21 days,
- cell growth medium with Human Platelet Lysate concentration decreased to 5% and with the addition of bFGF (0.1%; Gibco) for 10 days, then with the addition of retinoic acid (RA; Sigma-Aldrich) for the next 15 days,
- cell growth medium with the addition of N21 supplement (1:49; Sigma-Aldrich) for 21 days.

The second method of assessing ASC/DFAT ability to neural lineage differentiation was their co-culturing with neural tissue (organotypic hippocampal culture, OHC) or damaged neural tissue (OHC after oxygen–glucose-deprived, OGD). During this procedure, the cells at the 3rd passage were seeded on round glasses that were placed earlier in 24-well plates (Nunc, Thermo Fischer Scientific). After overnight incubation in humidified conditions under 5% O_2_, 5% CO_2_ at 37 °C, when the cells were adherent, glasses were placed under the membranes with hippocampal slices or damaged with OGD hippocampal slices. The ASC/DFAT were co-cultured with neural tissue in both variants of the experiment up to 7 days, then the cells and the hippocampal slices were analyzed with immunocytochemical methods or molecular biology.

### 2.9. Organotypic Hippocampal Slices Culture (OHC)

For this experiment 7-day-old Wistar rats from the Mossakowski Medicine Research Centre Animal Breeding House were used.

**Figure 4:**
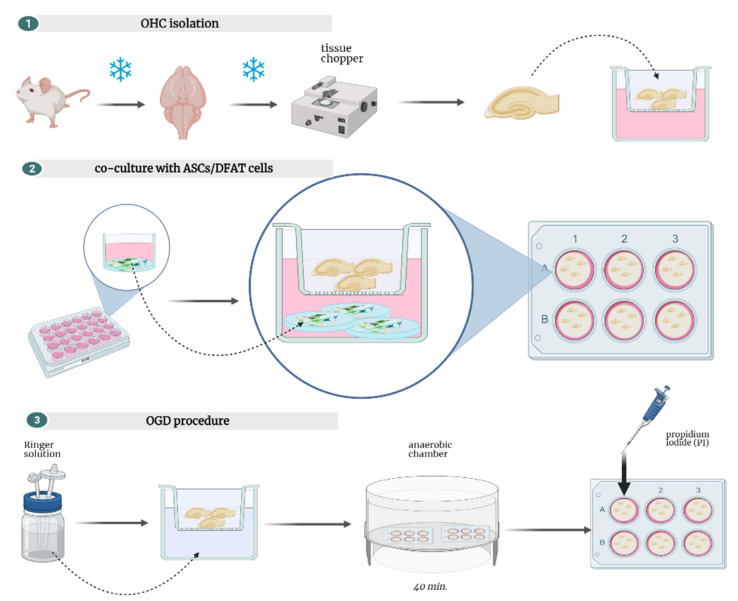
Steps of performed ex vivo experiments. The scheme was made in Biorender (https://biorender.com/, accessed date: 10 January 2021).

All procedures were made on ice. The previously described Stoppini’s method for obtaining organotypic hippocampal slices was modified in our lab [15]. After decapitation, brains were extracted, and hippocampi were isolated. Subsequently, the hippocampi were sliced for 400 µm slices with McIllwain’s tissue chopper (Ted Pella, Poznan, Poland) and were transferred on the membranes for organotypic culture (Millipore, Concord Road Billerica, MA, USA). The membranes were placed in 6-well dishes (Nunc, Thermo Fischer Scientific) (Figure 4); every well was filled with 960 µL of medium consisting of Neurobasal-A a(Gibco), 25% HBSS (Gibco), HEPES (Gibco), 5 mg/mL glucose (Sigma-Aldrich), 2 mmol/L L-glutamine (Gibco), B-27 supplement (Thermo Fisher Scientific) and antibiotic-antimycotic solution (Gibco). The medium was changed every 2/3 days and the cultures were carried out in 34 °C, 5% O_2_, 5% CO_2_.

### 2.10. Oxygen-Glucose-Deprivation (OGD)

On the 7th day of culture, the OHC was stained with propidium iodide (PI; Thermo Fisher Scientific). Labelled with PI (Sigma-Aldrich), damaged in cornu ammonis (CA)-region hippocampal slices were discarded. Then, after replacement of the medium with deoxygenated Ringer’s solution (Sigma-Aldrich) with mannitol (Sigma-Aldrich), the hippocampal slices (on the membranes) were transferred into an anaerobic chamber. The incubation was performed for 40 min. Then, the membranes were rinsed in PBS (Macopharma) 3 times and used to assess ASCs/DFAT cells’ neurogenic or neuroprotective potential.

Twenty-four hours after the OGD procedure, the hippocampal slices derived from the co-culture with the cells were stained with PI (Macopharma) for thirty minutes. Next, cell death quantification was performed. Images of hippocampal slices were acquired using a laser scanner microscope LSM 510 (Zeiss).

Relative cell death was calculated from each standardized CA region according to the following formula:

% of dead cells = (experimental fluorescent intensity (FI) − background FI)/(maximal FI − background FI) × 100.(1)

### 2.11. Immunocytochemistry

For immunocytochemical analysis, the cells at 3rd or 4th passage were seeded on 24-well plates (Nunc, Thermo Fischer Scientific) at 2.5 × 10^3^ cells/cm^2^ density. At 70% confluence, the cells were washed carefully in PBS (Macopharma), fixed with 4% PFA (Sigma-Aldrich) for 15 min at room temperature and washed in PBS (Sigma-Aldrich) again. An amount 0.2% Triton X-100 (Sigma-Aldrich) was used to permeabilize cell membranes in case of detecting intracellular target antigen. To block nonspecific binding, a mixture of 10% goat serum (Gibco) and 1% bovine serum albumin (Sigma-Aldrich) was applied for one hour. Subsequently, cultures were washed with PBS (Sigma-Aldrich) and incubated with primary antibodies (Table 2) for 24 h at 4 °C. For every variant of staining, negative control was performed to analyze the specificity of the reaction. On the following day, the cells were washed in PBS (Sigma-Aldrich), and the secondary antibodies (Table 3) were added in darkness for one hour. After the cells were washed with PBS (Sigma-Aldrich) again, the nuclei were stained with Hoeschst 33342 (Sigma-Aldrich) for 15 min.

The samples were analyzed with LSM 780 confocal laser scanning system and ZEN software (Carl Zeiss). Quantitative analysis was performed as a relation of positive cells to all cells (50 cells in one repetition). Each variant had 3 repetitions.

### 2.12. Three Germ Layer Differentiation Potential

The cells’ three-germ-layer differentiation potential was determined using components of a Human Pluripotent Stem Cell Functional Identification Kit (R&D Systems, Minneapolis, MN, USA), which is dedicated to the examination of iPSC. For DFAT cells assessment; glasses were coated by poly-L-lysine and media were compound with αMem (Macopharma) and bFGF (Gibco). After the 5th day of culture, cells were fixed using 4% PFA (Sigma-Aldrich). The results of differentiation were estimated based on immunocytochemical staining (according to the manufacturers protocol) and evaluation of OTX2, Brachyury, and SOX17 gene expression with RT-PCR in comparison with undifferentiated cells cultured without differentiating supplements as a control group.

### 2.13. Quantitative RT-PCR Analysis

The mRNA probes were isolated by fenozol according to the protocol instructions (A&A Biotechnology, Gdansk, Poland). The purity of each sample was measured by NanoDrop ND-1000 (Thermo Scientific, Thermo Fischer Scientific), then reverse transcription was conducted using a High-Capacity RNA-to-cDNA Kit (Applied Biosystems, Thermo Fischer Scientific). Quantitative RT-PCR reaction was performed by Fast 7500 Thermocycler (Applied Biosystems) with 10 ng of cDNA in 15 μL reaction mixture containing 3-color RT HS-PCR Mix SYBR^®^ (A&A Biotechnology), and 0.25 μM/μL for each specific primer. For stemness-related transcriptional factors, NANOG, SOX2, OCT3/4, and REX1 expression was estimated. For three-germ-layer differentiation, SOX17, OTX2, and Brachyury gene expressions were measured, and for OGD, GFAP, nestin, MAP2, S100beta, B-tubulin III, and NG2 gene expressions were measured (Table 4). Final gene expressions were calculated by the 2^-ΔΔCt^ method with β-actin as a reference gene.

### 2.14. Cytokine and Chemokine Assays with Luminex Kit

Eight-plex Human Magnetic Luminex Assays (R&D Systems, cat. no. LXSAHM-08) were used to measure the cytokine or protein concentration in medium samples. There were three variants of the medium collected: ASC/DFAT co-culture with OHC, ADSC/DFAT co-culture with OHC after OGD, and ASC/DFAT culture without OHC. Moreover, the basal medium without any cells was analyzed. The concentration of BDNF, FGF, HGF, beta-NGF, EGF, GDNF, LIF, and VEGF were analyzed using a Luminex-based platform and Luminex 200 IS V2.1 Software (Bio-Rad, Hercules, CA, USA). Standard curves were generated from the reference cytokine gradient concentrations. All samples were prepared in this same way; after the medium collection, samples were liquated and stored at −80 °C. All procedures of the media analysis were conducted on the ice. Each sample was frozen/thawed only once.

### 2.15. Statistical Analysis

The statistical analysis of the raw data was performed using GraphPad Prism 7 software. Data are presented as the mean and standard deviation. One-way analysis of variance (ANOVA test) was used to conduct multi-group comparisons, followed by Tukey test as post hoc statistical analysis for each group. The values were considered significant with *p* < 0.05.

## 3. Results

### 3.1. Multipotent Properties of Mesenchymal Stem Cells

**Figure 5:**
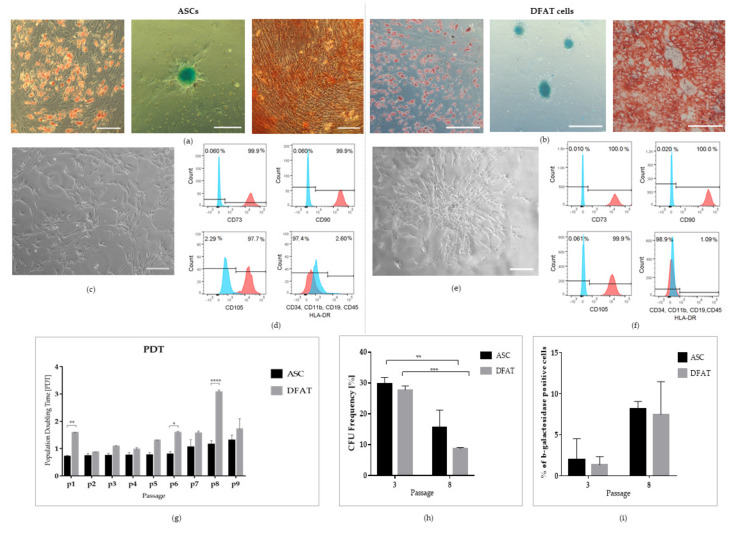
Multipotent properties of mesenchymal stem cells. (**a**)ASCs’ Differentiation into adipocytes, chondrocytes and osteoblasts at 2nd passage of culture in 21% O_2_ conditions; (**b**)DFAT cells differentiation into adipocytes, chondrocytes and osteoblasts at 2nd passage of coulture, in 21% O_2_ conditions; (**c**) Morphology of ASCs in 3rd day of culture, scale bar- 100 µm; (**d**) Flow cytometry analysis of ASCs’mesenchymal markers presence: CD73, CD90, CD105 and absence: CD34, CD11b, CD19, CD45 and HLA-DR at 2nd passage of culture; (**e**) Morphology of DFAT cells in 3rd day of culture, scale bar—100 µm; (**f**) Flow cytometry analysis of DFAT cells; mesenchymal markers presence: CD73, CD90, CD105 and absence: CD34, CD11b, CD19, CD45 and HLA-DR at 2nd passage of culture; (**g**) Population Doubling Time analysis from 1 to 9 passage; (**h**) Colony Formation Unit Frequency analysis at 3rd and 8th passage; (**i**) Senescence evaluation at 3rd and 8th passage. Cells cultured conditions: 5% O_2_, 5% CO_2_, 37 °C. The results are presented as mean values of three experiments ± SEM. The differences were considered statistically significant when *p*-value < 0.05. Statistical significance level * for 0.01 < *p* < 0.05; ** for 0.001 < *p* < 0.01; *** for 0.0001 < *p* < 0.001; **** for *p* < 0.0001.

After ASCs and DFAT cells’ isolation, phenotypic differences were observed. On the 3rd day of culture, both ASCs and DFAT cells showed the typical fibroblast-like phenotype, but ASCs were growing evenly on the plastic surface (Figure 5c), and the DFAT cells were grown as a rosette form with small oil vacuoles inside the cell’s matrix (Figure 5e). The release of lipid droplets was observed during the next couple of days of standard adherent culture. To assess the multipotent properties of cells, the ability of the cells to differentiate into adipocytes, chondrocytes, and osteocytes was tested. Mesodermal lineage differentiation potential was analyzed by culturing the cells with the addition of standard adipogenic, chondrogenic, and osteogenic medium supplements. Our results showed that DFAT cells can redifferentiate into adipose cells. The accumulation of lipid drops inside the cell’s matrix was observed, as evidenced by oil red staining after two weeks of culture. Cultivation of DFAT cells with the addition of osteogenic-induced supplement showed the presence of azalin red-stained calcium deposits after two weeks of culture. DFAT cells growing in the presence of chondrogenic differentiation induction medium took the shape of micromass pellets with positive alcain blue staining that suggests the accumulation of cartilage proteoglycans after 21 days of differentiation (Figure 5b). To assess the multipotent properties of cells, a standard flow cytometry analysis of surface antigen profiles was also performed. Both ASCs and DFAT cells showed high expression of mesenchymal cell surface antigens at 2nd passage of culture. Both populations were uniformly positive in CD73, CD105, and CD90 and negative in CD34, CD11b, CD19, CD45, and HLA-DR, with no significant differences between populations (Figure 5d,f). The long-term proliferation analysis was conducted by performing the population doubling time (PDT) analysis up to the 9th passage. The positive effect on the rate of cell proliferation remained at a similar level in both ASCs and DFAT cells and was approximately the same for eight passages. The number of population doubling did not differ significantly between populations except for 1, 6, and 8 passages, in which a longer population doubling time was observed in DFAT cells (Figure 5g). A clonogenity analysis was performed at the 3rd and 8th passage. Our results show that initially the clonogenity for both populations—ASCs and DFAT cells—were comparable; however, at passage 8 we observed significantly decreased values in ASCs from 30% ± 2 to 15% ± 6 and in DFAT cells from 28% ± 2 to 9% ± 1 (Figure 5h). The senescence analysis was also carried out at two time points: 3rd and 8th passage by using the β-galactosidase assessment. Our analysis showed the highest enzyme activity at the 8th passage in comparison with the 3rd passage. In ASCs, we observed a significantly higher percentage of β-galactosidase positive cells, which amounted 2% ± 2.5 at the 3rd passage in comparison with 8% ± 1 at the 8th passage. Moreover, in DFAT cells, a similar data dependency was demonstrated. We showed increased values of senescence positive cells from 1.3% ± 1 to 7.5% ± 4. (Figure 5i).

### 3.2. Pluripotent Properties of ASCs and DFATs

**Figure 6:**
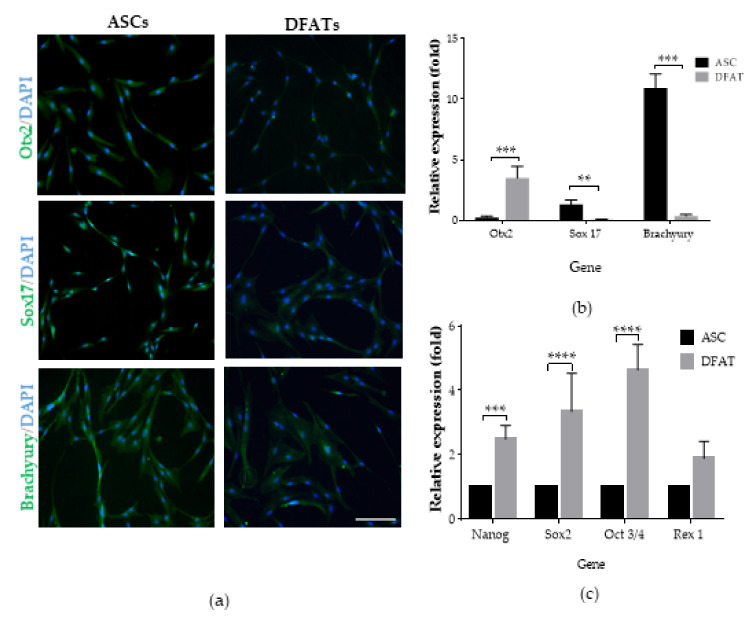
Pluripotent properties of ASCs and DFAT cells: (**a**) Immunocytochemical analysis of differentiation into ectoderm (Otx2), endoderm (Sox17), and mesoderm (Brachyury) at 2nd passage of culture. Scale bar = 100µm; (**b**) qRT-PCR analysis of 3-germ-layer gene expression: Otx2, Sox17, and Brachyury in 2nd passage with undifferentiated cells as a control group; (**c**) qRT-PCR analysis of stemness-related transcriptional factors (SRTF) at the 2nd passage of culture with ASCs as a control group. Cells cultured conditions: 5% O_2_, 5% CO_2_, 37 °C. The results are presented as mean values of three experiments ± SEM. The differences were considered statistically significant when *p*-value <0.05. Statistical significance level * 0.01 < *p* < 0.05; ** 0.001 < *p* < 0.01; *** 0.0001 < *p* < 0.001; **** *p* < 0.0001.

To examine the pluripotent properties of the cells, differentiation into three germ layers was evaluated. To confirm the ability to differentiate, immunocytochemical staining and qRT-PCR analysis of the specific markers’ expression was performed at the fifth day of cells differentiation. We assessed Otx2 gene expression as an ectodermal marker, Sox17 as an endoderm, and Brachyury as a mesoderm. Immunocytochemical staining confirmed the differentiative potential of both DFAT cells and ASCs because of the presence of nuclear antibody light signal (Figure 6a). Quantitative real-time PCR showed a different tendency and revealed highly significant increased values of the relative expression levels of the Otx2 gene in DFAT cells, which were detected in the level of 3.5 ± 1 compared with the ASCs’ levels of 0.2 ± 0.1. We also showed a significant high level of Brachyury gene expression, which was 11 ± 1 in ASCs in comparison with a significantly lower 0.4 ± 0.4 level of Brachyury expression in DFAT cells. Moreover, the Sox17 gene expression values were significantly higher during ASCs’ differentiation in endoderm 1.3 ± 0.4 in comparison with DFAT ability 0.1 ± 0. (Figure 6b). Our results suggest that DFAT cells have significantly greater abilities to differentiate into ectoderm cells than ASCs. Our findings also clearly demonstrate the advantage of ASCs in mesodermal and endodermal differentiation in comparison with DFAT cells’ abilities. To more accurately assess the pluripotent abilities of ASCs and DFAT cells, the expression of stemness-related transcriptional factors (SRTF) was assessed by the qRT-PCR technique. We assessed the expression level of the Nanog, Sox2, Oct 3/4, and Rex1 genes, which are important markers of pluripotency and are involved in the maintenance of pluripotent abilities such as ESCs. We observed that DFAT cells showed significantly higher values of the relative expression of all factors mentioned above and that expression values were approximately 2- to 5-fold higher compared with the expression levels in ASC. The Nanog gene relative expression was detected as 2.5 ± 0.4 and was significantly higher than ASCs Nanog expression values. A similar data dependency was observed when detecting the expression of the Sox2 gene, whose level was 3.4 ± 1.2, Oct ¾, where the relative expression was 4.6 ± 0.8, and Rex1, where the detected expression was 1.9 ± 0.5 compared with the expression of these genes in ASCs. (Figure 6c.).

### 3.3. Optimization of ASCs Capacity for Neural Lineage Differentiation

**Figure 7:**
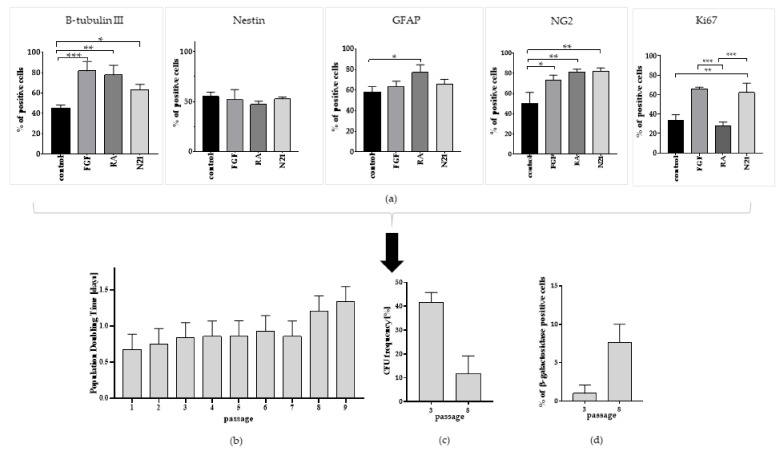
Optimization of ASCs’ capacity for neural lineage differentiation. (**a**) Quantitative analysis of neural (B-tubulin III, Nestin, GFAP, NG2) and proliferation (Ki67) markers presence in ASC culture in inducive of neurogenesis medium (with addition of FGF, N2, N21); (**b**) Estimation of Population Doubling Time of ASC culture in medium with N21 supplement; (**c**) Colony Forming Unit at early (3rd) and late (8th) passage of ASC culture supplemented with N21. (**d**) Percentage of senescent cells counted from early (3rd) and late (8th) passage of ASC supplemented with N21. The results are presented as mean values of three experiments ± SEM. The differences were considered statistically significant when *p*-value <0.05. Statistical significance level * for 0.01 < *p* < 0.05; ** for 0.001 < *p* < 0.01; *** for 0.0001 < *p* < 0.001; **** for *p* < 0.0001.

To optimize the conditions for the highest neurogenic ASCs’ potential, a quantitative analysis of the selected neural markers and a marker of proliferation presence was performed. The results of immunofluorescence staining showed significantly higher levels of B-tubulin III and NG2-positive cells among the ASCs cultured with stimulating factors (Figure 7). The highest percentage of neural marker B-tubulin III+ cells was found in the population grown in bFGF medium, oscillating to 82 ± 9. In both RA- and N21-supplemented medium culture variants, the percentage of oligodendrocytic marker NG2+ cells was high and oscillated to 81% ± 3. The GFAP astrocytic marker showed its highest level for the cells in RA-supplemented medium (77% ± 8). These results were significantly different from ASCs grown in standard control medium, where its value was about 58% ± 6. Immunofluorescent staining pictures are presented in Supplementary Materials (Figure S1). The number of nestin+ cells was comparable in every variant. The high marker of proliferation Ki-67 presence was maintained during N21- and bFGF-supplemented ASCs cultures. Although the results obtained from differentiation performed with N21 and bFGF supplementation in the aspect of oligodendrocytic and proliferation quantitative marker analysis was similar, we observed morphological differences in the culture. As a result of ASCs differentiation with N21 supplement, the spindle shape was maintained throughout the whole experiment. After 10 days of cultivation, the presence of small cells was still observed, but elongated, spindle-shaped cells prevailed. During cultivation in the medium supplemented with bFGF, the cells firstly did not elongate but kept the unchanged spindle shape. However, after 10 days, the cells began to expand, lengthen, and flatten more intensively. The change of their shape could have been related to a higher rate of senescence; thus, we decided to use N21 supplement in further studies. The assessment of the most efficient conditions inducing neural differentiation, including the high presence of selected neural markers and proliferation potential, suggested that N21 supplemented ASCs’ culture variant as the most beneficial in comparison with other variants. Following these results, the population doubling time, colony forming unit, and the number of senescent cells in N21 supplemented ASCs culture were analyzed. There was no significant influence between these variant results and the control culture of ASCs throughout the passages (Figure 5 and Figure 7).

### 3.4. Comparison of ASCs and DFAT Cells Neural Differentiation Capacity

**Figure 8:**
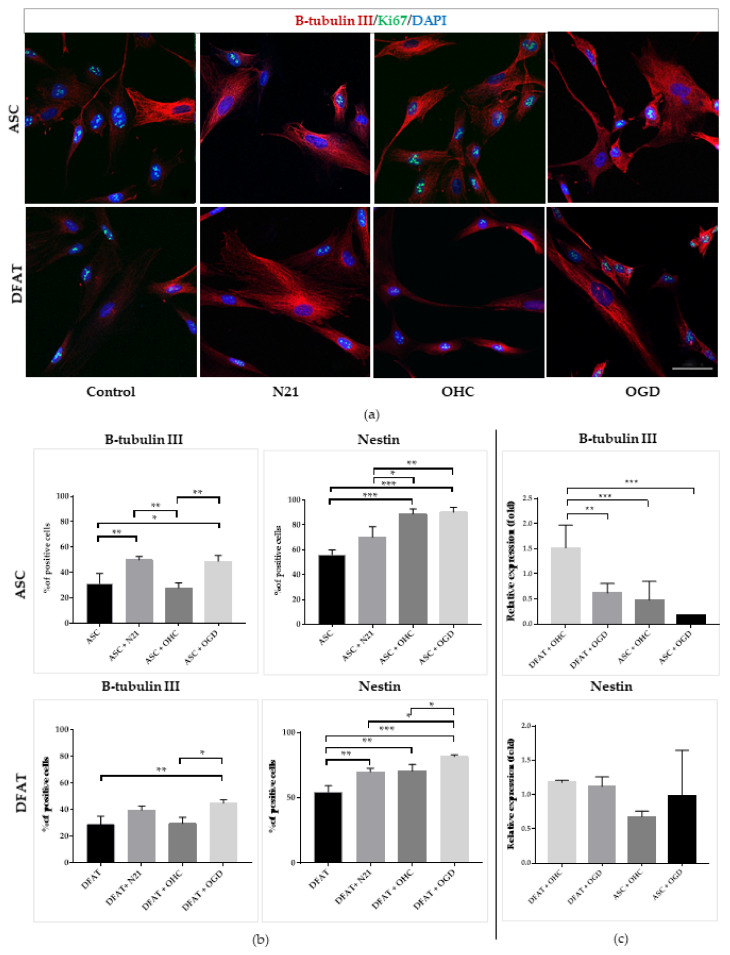
Neural differentiation of ASCs/DFAT cells. B-tubulin III, Nestin immunocytochemical/expression analysis. (**a**) Immunofluorescent images of ASCs/DFAT cells B-tubulin III staining; (**b**) Quantitative analysis of neural markers presence in different culture conditions; (**c**) Neural markers (B-tubulin III and Nestin) expression. The results are presented as mean values of three experiments ±SEM. The differences were considered statistically significant when *p*-value < 0.05. Statistical significance level * for 0.01 < *p* < 0.05; ** for 0.001 < *p* < 0.01; *** for 0.0001 < *p* < 0.001; **** for *p* < 0.0001.

Immunocytochemical and gene expression analysis of early neural markers demonstrated the valid impact of neural tissue presence on ASCs and DFAT cells’ neural differentiation capacity, especially after damage (Figure 8). The level of nestin+ cells was significantly increased in two types of the examined cells co-cultured with OHC after the OGD procedure in comparison with the culture lead in control conditions, reaching 90% ± 4 for ASCs and 81% ± 1 for DFAT cells. Interestingly, the gene expression analysis indicated the converse results, where DFAT cells showed stronger expression of early neural markers in both ex vivo variants in comparison with the ASCs. After the in vitro culture, by immunofluorescence analysis, we also confirmed that N21 supplement was significantly increasing the level of B-tubulin III- and nestin-positive cells for both cell types, although a greater effect in the case of nestin marker presence was observed in ASCs culture. B-tubulin III marker levels were comparable in both ASCs and DFAT cells culture lead with N21 supplement and in OHC after OGD co-culture.

**Figure 9:**
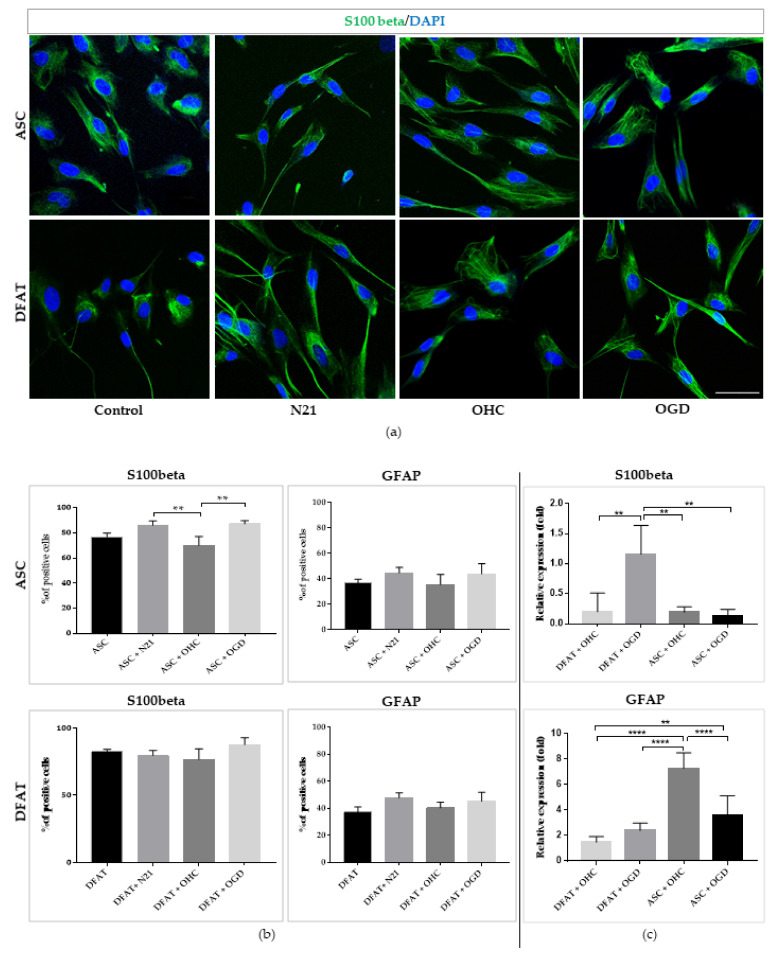
Astrocytic differentiation of ASCs/DFAT cells. S100beta, GFAP immunocytochemical/expression analysis. (**a**) Immunofluorescent images of ASCs/DFAT cells S100beta staining; (**b**) Quantitative analysis of astrocytic markers (GFAP, S100beta) presence in different culture conditions. (**c**) Astrocytic markers expression. The results are presented as mean values of three experiments ± SEM. The differences were considered statistically significant when *p*-value <0.05. Statistical significance level * for 0.01 < *p* < 0.05; ** for 0.001 < *p* < 0.01; *** for 0.0001 < *p* < 0.001; **** for *p* < 0.0001.

Regarding the assessment of differentiation into astrocytes’ ability, after the immunocytochemical staining, we estimated a comparable high percentage of S100beta and GFAP positive cells in every ASC and DFAT culture variant (Figure 9). However, in ASCs culture, we determined a significant difference in S100beta+ cells between the ASCs co-cultured with OHC and with OHC after OGD, which was greater and similar to ASCs supplemented with N21, which oscillated to 87% ± 3. Astrocytic gene expression in ex vivo culture indicated a very strong expression of S100beta in DFAT supplemented with N21 in comparison with other variants, whereas GFAP was conversely strongly expressed by ASCs after OHC after OGD co-culture.

**Figure 10:**
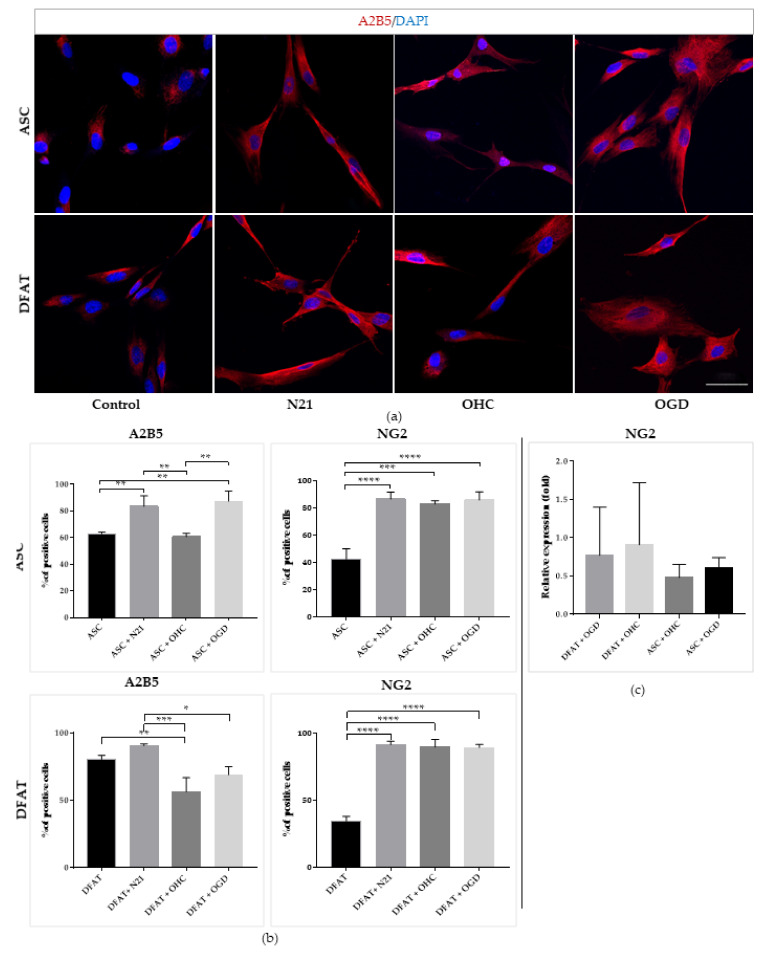
Oligodendrocytic differentiation of ASCs/DFAT cells. A2B5,NG2 immunocytochemical/expression analysis. (**a**) Immunofluorescent images of ASCs/DFAT cells A2B5 staining; (**b**) Quantitative analysis of oligodendrocytic markers (A2B5, NG2) presence in different culture conditions; (**c**) Oligodendrocytic marker NG2 expression. The results are presented as mean values of three experiments ± SEM. The differences were considered statistically significant when *p*-value <0.05. Statistical significance level * for 0.01 < *p* < 0.05; ** for 0.001 < *p* < 0.01; *** for 0.0001 < *p* < 0.001; **** for *p* < 0.0001.

Moreover, we applied immunocytochemistry to examine the percentage of oligodendrocytic markers, A2B5 and NG2-positive cells (Figure 10). The results showed a similarly high impact of N21 supplement and OHC after OGD co-culture on the amount of A2B5+ ASCs. Strong significant differences were observed for NG2 marker presence, which was strongly increased for both ASCs and DFAT cells’ N21-supplemented and ex vivo culture variants in relation to the control, reaching around 2 times higher values, more than 80%. On the molecular level, DFAT cells displayed a stronger expression of the NG2 marker than ASCs in both ex vivo variants.

**Figure 11:**
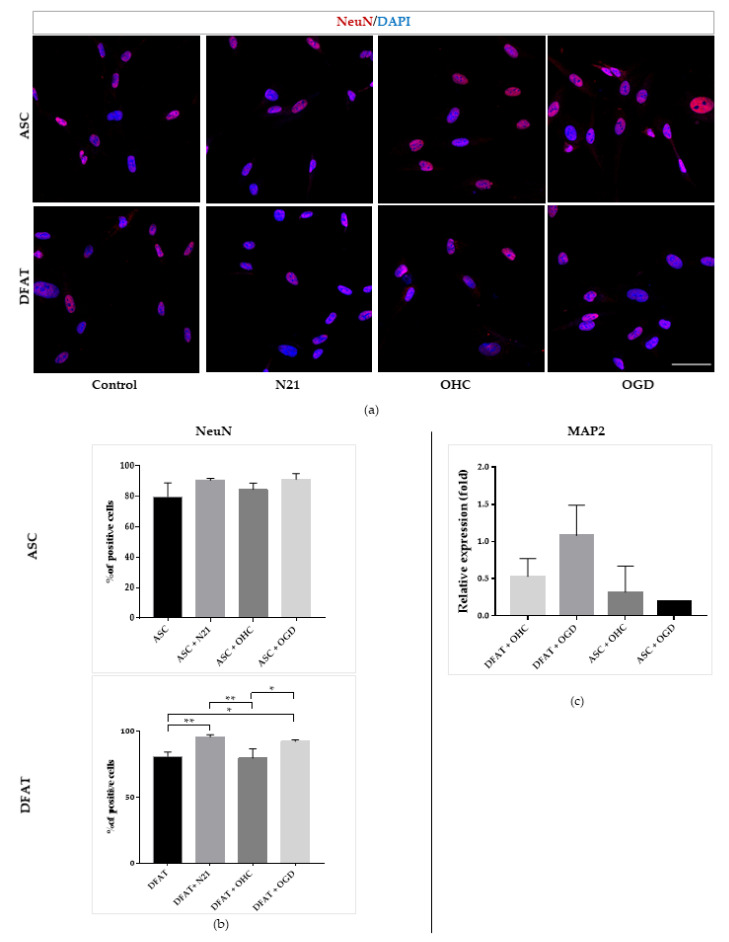
Neuronal differentiation of ASCs/DFAT cells. NeuN, MAP2 immunocytochemical/expression analysis. (**a**) Immunofluorescent images of ASCs/DFAT cells NeuN staining; (**b**) Quantitative analysis of neuronal marker NeuN presence in different culture conditions; (**c**) Neuronal marker MAP2 expression. The results are presented as mean values of three experiments ± SEM. The differences were considered statistically significant when *p*-value <0.05. Statistical significance level * for 0.01 < *p* <0.05; ** for 0.001 < *p* < 0.01; *** for 0.0001 < *p* < 0.001; **** for *p* < 0.0001.

Finally, we examined the neuronal differentiation ability of the cells using immunocytochemical analysis of NeuN marker and the expression of MAP2 (Figure 11). Immunofluorescent images confirmed the high level of NeuN in each cell variant. It was significantly increased in DFAT cells after N21 supplementation and OHC after OGD co-culture than in that of the control and after OHC co-culture. In the ex vivo model, MAP2 expression was relatively high in DFAT cells, especially after co-culture with OHC after OGD procedure.

### 3.5. Neuroprotective Abilities of ASCs and DFAT Cells in the Ex Vivo Model

**Figure 12:**
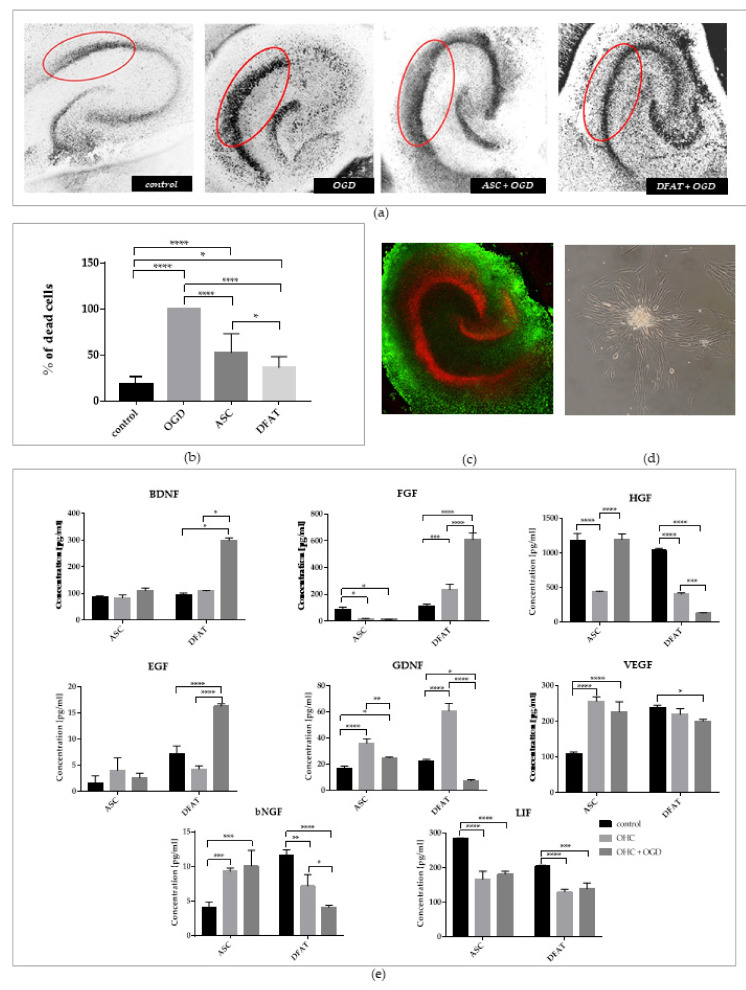
ASCs/DFAT cells neuroprotective and neurogenic potential assessment. (**a**) PI incorporation into the CA1 region of the hippocampal slices (the region is marked red); (**b**) Quantitative analysis of dead cells after OGD in all culture variants.; (**c**) Immunocytochemical analysis of live (green—stained with Calcein) and dead (red—stained with PI) cells of the hippocampal slice; (**d**) The characteristic rosette made by ASCs after the co-culture with OHC; (**e**) Cytokines concentration in medium collected in three culture variants—from cell culture (control), after co-culture with organotypic hippocampal slices (OHC) and with OHC after oxygen-glucose deprivation (OHC+OGD).The results of all presented above experiments are expressed as mean values of three experiments ± SEM. The differences were considered statistically significant when *p*-value < 0.05. Statistical significance level * for 0.01 < *p* < 0.05; ** for 0.001 < *p* < 0.01; *** for 0.0001 < *p* < 0.001; **** for *p* < 0.0001.

In this study, we assessed the impact of ASCs/DFAT cells’ indirect co-culture with organotypic hippocampal slices after OGD on their neuroprotective properties (Figure 12). The survival of hippocampal cells in the CA1 region after OGD was evaluated after the analysis of PI (propidium iodide, a marker of dead cells) incorporation. We observed significantly lower mortality of hippocampal cells after the co-culture with both ASCs/DFAT cells. However, the neuroprotective effect induced by DFAT cells was significantly increased in comparison with ASCs, with the dead cells of hippocampi approximately reaching 36.4% ± 11.8. Moreover, the levels of the examined neutrophic factors were significantly higher in DFAT cells than in ASCs culture in the case of BDNF, FGF, and EGF, especially after co-culture with OHC after OGD. In the case of LIF, a decreased level was seen for both types of cells after co-culture.

## 4. Discussion

In this study, we mainly focused on the characterization of the neurogenic and neuroprotective abilities of the cell subpopulation isolated from adipose tissue—dedifferentiated adipocyte-derived progeny cells (DFAT cells). We performed the effective isolation using the ceiling culture and determined the basic parameters and properties of the cells compared with the standard ASCs.

Our results regarding the essential characteristics of DFAT cells are in line with previous reports [15]. We showed that DFAT cells present the same cell surface antigens profile as ASCs. There were no significant differences between the population doubling time, proliferation capacity, clonogenity, or senescence properties in a long-term culture. The multilineage potential and differentiation into adipocytes, chondrocytes, and osteocytes in both fractions also remained similar, whereas the expression of stemness-related transcriptional factors (SRTF)—Sox2, Nanog, Oct3/4, and Rex1—testified to the higher pluripotent capacity in DFAT cells, which is consistent with previous several independent studies [45,46,47]. Studies by Jumabay et al. confirmed the expression of pluripotent markers Oct3/4, SOX2, Nanog, c-Myc, and Klf4 and the ability to create clusters in addition expressing of markers characteristic of embryonic stem cells SSEA-1 and SSEA-3. All these features were much more strongly expressed in DFAT cells than ASCs; interestingly, the highest expression of pluripotent genes was noted in the fifth day of culture (5 DIV) using the membrane method and then decreased up to 20 days of cultivation. These results indicate the transient pluripotent nature of DFAT cells. We analyzed the cells in passage 2 (about 5–7 DIV, starting from day 0, which was the day ceiling cultivation ended).

Except for the expression of SRTF genes, the distinct pluripotent properties could also be indicated by the cell ability to differentiate toward the three germ layers: mesoderm, ectoderm, and endoderm.

After 5 days of culture ASCs has been shown to significantly higher express Brachyury gene, which is a marker of mesodermal differentiation, than DFAT cells. On the other hand, DFAT cells demonstrated stronger expression of the Otx2 gene, which is proved to be related to ectodermal differentiation. Our results differ from those published so far [45], as our findings show that DFAT cells do not have the ability to differentiate into all three types of germ layer cells but only into ectoderm. These discrepancies may be associated with the cell migration during epithelial–mesodermal transition (EMT) that was described earlier So far, the neural crest origin of a certain fraction of ASCs has been found, while in the case of DFAT cells such reports do not exist [10]. Our results may therefore suggest that the origin of the DFAT cell fraction may be related to neural crest migration, which may confirm their pluripotent nature, but that further studies for this purpose should be conducted.

Another possibility is that adipose tissue, similar to bone marrow themselves, contain an infinitesimal number of pluripotent cells and that the ceiling culture technique (as the stress factor) allows the isolation of that subpopulation. The technique of ceiling culture was described as a way of obtaining a more homogeneous and a less contaminated population of cells without, e.g., smooth muscle cells or fibroblasts compared with ASC [13]. It should be noted that the technique, with membrane filter and one-day incubation of adipocytes in suspension, allows an analysis to additionally exclude contamination with other cells, the adhesion of which is forced in the ceiling culture [45]. The conclusion is that subsequent modifications of the method for isolating DFAT cells allows the fraction of cells with stronger pluripotent properties to be obtained as well as providing the potential to differentiate cells into germ layers other than mesoderm [48]. Perhaps hypothetically, the isolation method used to obtain DFAT allows the fraction of cells derived from the neural crest and settled in the adipose tissue to be obtain.

Nevertheless, demonstrating the origin and the pluripotent nature of DFAT cells in vitro depends on many variables; therefore, determining an efficient method for obtaining these cells and for determining the conditions and time of culture requires separate and broader studies.

Mesenchymal stem/stromal cells mostly require additional environmental factors to differentiate into the neural direction. The selection of proper stimulating factors or even a mix of factors for supplementing the culture medium seems to be one of the most important components. Our study confirmed the beneficial effect of bFGF, RA, and N21 supplement presented in the medium on the ability of ASCs to differentiate into the neural direction. Naïve ASCs, without any additional pretreatment, were described to express nestin. We observed similar levels of nestin regardless of the current differentiating factor. The cells after bFGF treatment showed a significant high level of B-tubulin III, which is early neural marker, also considered by several authors as a marker of neurons [49,50,51]. Moreover, bFGF-treated ASCs indicated a high percentage of Ki-67-positive cells, a marker of proliferation. These observations are similar to those in the literature, conducted on the mouse bone marrow stromal cells [52]. ASCs not only expressed early neural markers but also astrocytic and oligodendrocytic markers after both RA and N21 treatment. It has been shown that the pre-activation of retinoid signaling by RA improves neuronal differentiation. However, the function of RA is also linked with the regulation of the proliferation—RA halts proliferation. What is more, it has been shown that the loss of RA signaling is linked with dedifferentiation and tumorigenesis [43]. The last cited study’s data are consistent with our findings. Although we observed a relatively high presence of neural and glial markers after RA treatment, there was a significant decrease of the percentage of Ki-67+ cells in comparison with other culture variants. To keep the high proliferative potential the additional factors should possibly be added, e.g., EGF and bFGF [53].

After N21 supplementation, we observed an unequivocally positive effect enhancing the neurogenic and proliferative potential of ASC; therefore, for further experiments with DFAT neurogenic differentiation, we choose that supplementation. The N21 supplement is described as the re-defined and modified supplement B27, for use in neuronal cultures [54]. DFAT cells responded to that supplementation, keeping their proliferative potential and expressing neural markers (neuronal, astroglial, and oligoglial). We have proved that DFAT cells have high potential for differentiating into neuronal-like cells in the presence of N21. The neural differentiation abilities of DFAT cells were also examined by Ohta et al. In the mentioned study, DFAT cell expression and protein levels of neural (β-tubulin III, nestin) and glial (GFAP) markers were confirmed [15].

Another question we wanted to answer was regarding the fate of DFAT in the presence of intact or injured neural tissue and without any additional exogenous stimulants. Previous studies have shown that ASCs have the ability to differentiate into the neural lineage (expression of neuronal markers) under the influence of organotypic hippocampal slices co-culture. Our observations regarding DFAT cells and ASCs differentiation are consistent with the results described by Sarnowska et al., who demonstrated the induction of neural differentiation of MSC in the absence of any additional chemical compounds or growth factors—differentiation that was only induced by co-culture with the neural tissue [55]. In our study, we observed significantly higher expression of early neural, astrocytic, oligodendrocytic, and neuronal genes in DFAT cells, especially after co-culture with the injured, OGD-treated hippocampal slices. Whereas, it was more strongly expressed by ASCs only regarding the expression of the GFAP astrocytic marker.

The therapeutic effect of MSC, however, is mainly related to its adjuvant properties. The secretory properties of ASCs may be enhanced and/or modulated by environmental factors, e.g., in response to the tissue injured or affected by a disease. Our field of interest is in the treatment of CNS diseases, where the therapeutic effect could be potentially related to the secretion of neuroprotective factors that could lower the scale of damage by reducing cell death [35,56]. The secretory properties of MSC may be enhanced and/or modulated by environmental factors, e.g., in response to injured tissue.

In our experiments, we observed an increased level of BDNF secretion, especially by DFAT cells co-cultured with OHC after OGD injury. BDNF is demonstrated as a candidate for defense candidate against ischemic brain injury [57,58,59,60,61,62]. It was shown, using animal model, that ASCs stimulate regeneration by secreting BDNF [63]. Moreover, the results indicated significantly stronger secretion regarding DFAT cells cultured with OHC and/or OHC after OGD than for other neurotrophic factors—FGF, EGF, and GDNF, previously described by several authors regarding ASCs’ secretome [64]. Our results showed a significant increase in the secretion of HGF and VEGF. Interesting differences were observed according to bNGF level changes, which were opposite those observed in ASCs in comparison with DFAT cells. Regarding ASCs, our findings were consistent with Sarnowska et al., who observed a significant increase of NGF expression by BM-MSC after co-culture with rat OHC after OGD [65]. Furthermore, the authors underlined the importance of culture conditions’ influence on cell neuroprotective abilities. The results obtained by Tan and co-workers showed the presence of NGF in ASCs’ cultured media and suggest that in addition to its neuroprotective properties, the medium mediates damaged tissue repair through the induction of neurogenesis via activation of NGF-induced AMP-activated kinase (AMPK) [66]. Accordingly, it was evaluated that human adipose mesenchymal stem cells can protect against glutamate-induced injury in P12 cells via the secretion of VEGF, HGF, BDNF, and NGF, both under normoxic and hypoxic conditions [67]. We also observed a significant decrease of LIF secretion in both populations of cells co-cultured with intact or injured hippocampal slices. This observation could be related to the progressive differentiation of the cells in the presence of neural tissue. Our observations regarding differences in cell secretion within different cell subpopulations in restricted culture conditions are consistent with those of Crigler and coworkers [68]. Their findings demonstrated that transcripts expressed by MSCs for BDNF and bNGF are strictly correlated with specific subpopulations, encoding axon guidance, neurite-inducing, and neural-cell-adhesion molecules. Interestingly, the researchers presumed that MSC-induced effects express factors other than neurotrophins, contributing to the above-mentioned activities. Moreover, the factor subset is co-expressed with BDNF in MSC subclones. In addition, the expression levels of BDNF are linked with the ability of cell populations/subclones to promote neuronal cell survival and neuritogenesis. It was also shown that MSCs promote neurite outgrowth within dorsal root ganglion explants despite secreting a 25-fold lower level of bNGF, which is required to produce similar effects exogenously. The secretion of several factors by ASCs and DFAT cells is influenced by the injured neural tissue. Moreover, in the case of both cell types as well as both co-culture variants that we presented in this study, treatment not only led to induced neuroprotection according to significantly increased secretory properties of the cells but also led to our observation of a reduced number of dead cells in the damaged nervous tissue after indirect co-culture, thus confirming the paracrine neuroprotective effect of both ASCs and DFAT cells, similar to results described previously for WJ-MSC and for BM-MSC [65,69].

The cells have a protective effect on the injured tissue, and conversely, the injured tissue affects the cells’ differentiation ability [34]. These dependencies complement each other and help to illuminate the phenomenon of MSCs’ therapeutic effectiveness, indicating the great importance of the attempts to use these cells in the regeneration of ischemically injured nerve tissue.

The impact of the damaged nerve tissue and transplanted cells is bilateral. In addition to secreted cytokines and growth factors, the affected tissue also secretes factors stimulating the cells to proliferate and differentiate into neural lineage. The pro-neurogenic properties of these factors lead to the activation and enhancement of endogenous neurogenesis, and thus may lead to the creation of an effective therapy for neurological disorders.

In conclusion, our results indicate that DFAT, the next subpopulation of stem/stromal derived from adipose tissue, is more homogenous, has slightly different secretory properties in relation to SVF and ASCs, and expresses some pluripotent-like features and strong neuroprotective properties. Moreover, in the presence of neural tissue, these cells differentiate toward neural direction without any additional stimulants.

As far as we know, this is the first paper presenting an analysis of the neurogenic and neuroprotective potential of both ASCs and DFAT cells.

## Figures and Tables

**Table 1:** Histochemical dyes (Sigma-Aldrich) used in differentiation analysis.

Differentiation Lineage	Used Stain	Visualized Compound	Time of Incubation (Minutes)	Concentration
**Adipogenesis**	Oil Red O	Oil Red	5	0.5%
**Osteogenesis**	Alizarin Red S	Calcium deposits	3	2%
**Chondrogenesis**	Alcian blue	Mucopolysaccharides	30	1%

**Table 2:** Primary antibodies used in immunocytochemistry analysis.

Antibody	Catalogue Number	Source	Isotype	Dilution	Manufacturer
**anti-β-Tubulin III**	T8660	Mouse monoclonal	IgG2b	1:1000	Sigma-Aldrich
**anti-GFAP**	Z0334	Rabbit polyclonal	IgG	1:400	Dako
**anti-Ki67**	AB15580	Rabbit polyclonal	IgG	1:200	Abcam
**anti-Nestin**	MAB5326	Mouse monoclonal	IgG1	1:500	Millipore
**anti-Fibronectin**	F3648	Mouse monoclonal	IgG	1:400	Sigma-Aldrich
**anti-Vimentin**	AB1620	Mouse monoclonal	IgG1	1:200	Abcam
**anti-NeuN**	MAB377	Mouse monoclonal	IgG1	1:50	Millipore
**anti-A_2_B_5_**	MAB312R	Mouse monoclonal	IgM	1:200	Millipore
**anti-NG_2_**	AB5320	Rabbit polyclonal	IgG	1:150	Millipore
**anti-S100 beta**	AB52642	Rabbit polyclonal	IgG	1:100	Abcam

**Table 3:** Secondary antibodies used in immunocytochemistry analysis.

Antibody	Fluorochrome	Catalogue Number	Isotype	Dilution	Manufacturer
**Alexa Fluor Goat** **(anti-rabbit)**	Alexa 546	A11035	IgG	1:1000	Life Technologies
**Alexa FluorGoat** **(anti-mouse)**	Alexa 546	A21123	IgG1	1:1000	Thermo Fisher Scientific
**Alexa Fluor Goat** **(anti-mouse)**	Alexa 488	A21121	IgG1	1:1000	Life Technologies
**Alexa Fluor Goat** **(anti-mouse)**	Alexa 488	A21141	IgG2b	1:1000	Life Technologies
**Alexa Fluor Goat** **(anti-mouse)**	Alexa 546	A21045	IgM	1:1000	Life Technologies

**Table 4:** Primer sequences used in qRT-PCR analysis.

Gene	NCBI Reference Sequence	Product Size	Primer Sequence (5e -> 3-)
**β-Actin**	NM_001101.5	250 bp	F: CATGTACGTTGCTATCCAGGC R: CTCCTTAATGTCACGCACGAT
**Nanog**	NM_024865.4	103 bp	F: GAACCTCAGCTACAAACAGG R: CGTCACACCATTGCTATTCT
**Sox2**	NM_003106.4	93 bp	F: GTGGAAACTTTTGTCGGAGA R: TTATAATCCGGGTGCTCCTT
**Oct3/4**	NM_001285986.2	331 bp	F: CCTGAAGCAGAAGAGGATCACC R: AAAGCGGCAGATGGTCGTTTGG
**Rex1**	NM_001304358.2	107 bp	F: GCTCCCTTGAATGTTCTTTG R: GCCTGTCATGTACTCAGAAT
**Oxt2**	NM_001270523.2	98 bp	F: TTCATGCGAGAGGAGGTGGCA R: TGCTGTTGTTGGCGGCACTT
**Sox17**	NM_022454.4	110 bp	F:AACTATCCTGACGTGTGACA R:CAAAAACCCAGGAGTCTGAG
**Brachyury**	NM_001379200.1	104 bp	F:ACGGCCACATTATTCTGAAT R:GAAGTTCTCCTCGGCATATT
**Nestin**	NM_006617.2	64 bp	F: GGGAAGAGGTGATGGAACCA R: AAGCCCTGAACCCTCTTTGC
**GFAP**	NM_001363846.2	100 bp	F: CCGACAGCAGGTCCATGT R: GTTGCTGGACGCCATTG
**MAP-2**	NM_001375545.1	99 bp	F: TTGGTGCCGAGTGAGAAGA R: GTCTGGCAGTGGTTGGTTAA
**β-Tubulin III**	NM_001197181.2	126 bp	F: GGAAGAGGGCGAGATGTACG R: GGGTTTAGACACTGCTGGCT
**S100beta**	NM_006272.3	91 bp	F: AGCGCTCCTGGAAAAAGCAA R: TTGAATCGCATGGGTCACGG
**NG2**	NM_001897.5	118 bp	F: GTCTACACCATCGAGCAGCC R: TGTGTGAGAACAGCACGAGC

## Data Availability

Not applicable.

## Supplementary Materials

The following are available online at https://www.mdpi.com/article/10.3390/cells10061475/s1, Figure S1: Immunofluorescent images of ASCs after bFGF, RA and N21 treatment.
