# Supplementary material for: Neurogenic and Neuroprotective Potential of Stem/Stromal Cells Derived from Adipose Tissue
Source: Cells. 2021 Jun 11;10(6):1475. doi: 10.3390/cells10061475 (PMC8231154; doi:10.3390/cells10061475)
Supplement: Supplementary file 1 [file cells-10-01475-s001.zip › cells-1210470-supplementary.pdf]

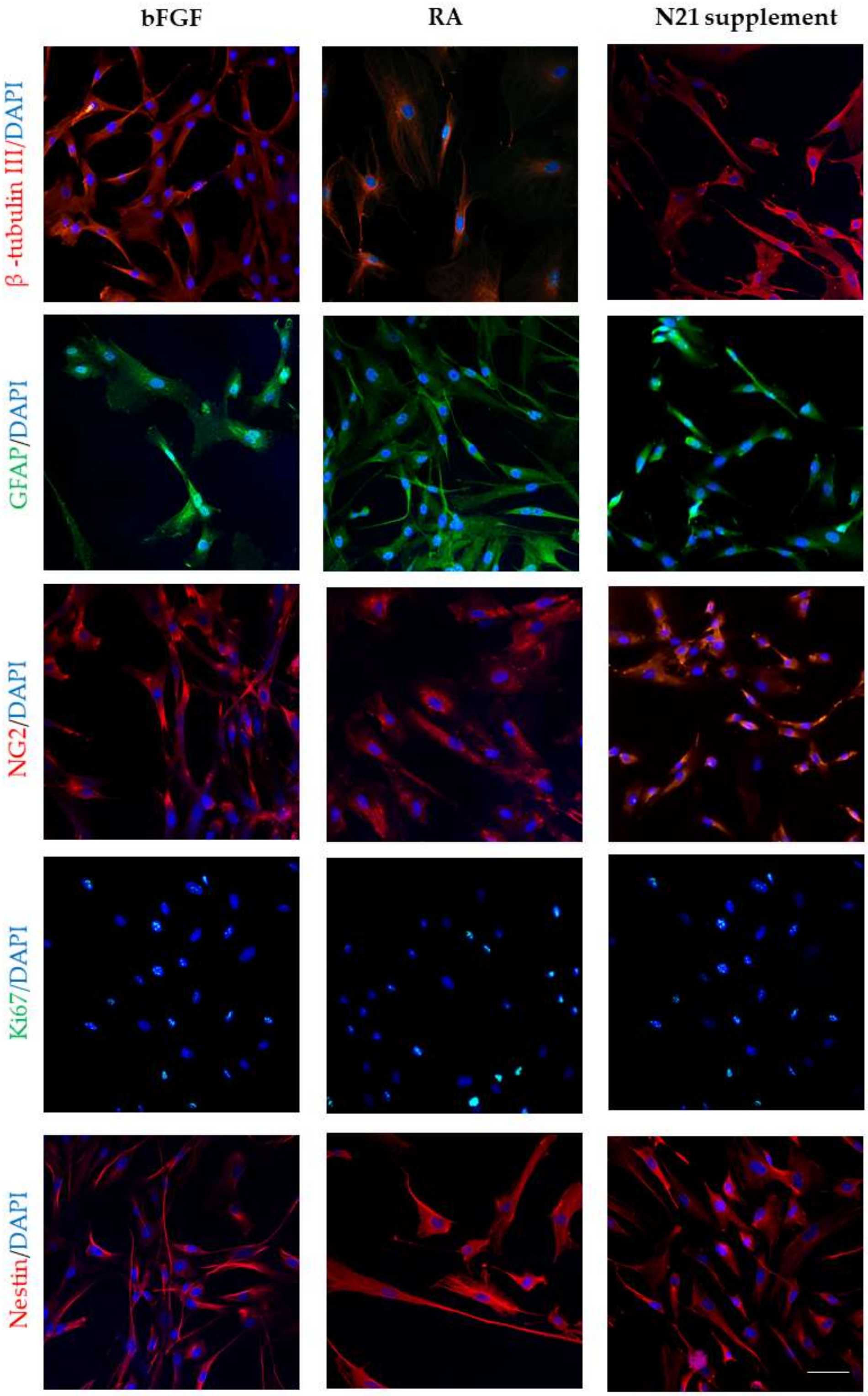

Figure S1. Immunofluorescent images of ASCs after bFGF, RA and N21 treatment. Stainings with neural (Nestin, NG2, GFAP, β -tubulin III), proliferation (Ki67) markers. Scale bar 50 μm.
